# Association between dietary inflammatory and antioxidant potential and systemic inflammatory and oxidative status with the risk and severity of coronary artery disease

**DOI:** 10.1371/journal.pone.0325716

**Published:** 2025-06-17

**Authors:** Zahra Namkhah, Elham Alipoor, Mahnaz Salmani, Negar Ebrahimi, Monireh Ahmadpanahi, Ali Vasheghani-Farahani, Mehdi Yaseri, Michael D. Wirth, Longgang Zhao, James R. Hebert, Mohammad Javad Hosseinzadeh-Attar

**Affiliations:** 1 Department of Nutrition, Faculty of Medicine, Mashhad University of Medical Sciences, Mashhad, Iran; 2 Department of Clinical Nutrition, School of Nutritional Sciences and Dietetics, Tehran University of Medical Sciences, Tehran, Iran; 3 Cardiac Primary Prevention Research Center, Cardiovascular Diseases Research Institute, Tehran University of Medical Sciences, Tehran, Iran; 4 Department of Clinical Cardiac Electrophysiology, Tehran Heart Center, Tehran University of Medical Sciences, Tehran, Iran; 5 Department of Epidemiology and Biostatistics, School of Public Health, Tehran University of Medical Sciences, Tehran, Iran; 6 College of Nursing, University of South Carolina, Columbia, South Carolina, United States of America; 7 Department of Epidemiology and Biostatistics and the Cancer Prevention and Control Program, University of South Carolina, Columbia, South Carolina, United States of America; 8 Department of Nutrition, Connecting Health Innovations, LLC, Columbia, South Carolina, United States of America; 9 School of Nursing, Yale University, New Haven, Connecticut, United States of America; Florida International University, UNITED STATES OF AMERICA

## Abstract

**Background and aims:**

Unhealthy diets have pro-inflammatory properties that have been shown to contribute to coronary artery disease (CAD). The dietary inflammatory index (DII^®^) and the dietary antioxidant quality score (DAQS) quantify the anti-/pro-inflammatory and antioxidant potential of a diet. This study aims to investigate the association between the energy-adjusted DII (E-DII^TM^), DAQS, oxidant/anti-oxidant biomarkers, and CAD risk and severity.

**Methods and results:**

This cross-sectional study investigated 158 participants for the presence and severity of CAD based on coronary angiography. E-DII and DAQS scores, malondialdehyde (MDA), total oxidant status (TOS), glutathione peroxidase (GPX) activity, total antioxidant capacity (TAC) and conventional cardiometabolic risk factors were assessed. The triglyceride-glucose (TyG) index and mean arterial pressure (MAP) were also calculated.

No association was observed between the E-DII or DAQS and the presence or severity of CAD. Based on the final regression models, age (adjusted odds ratio (AOR) =1.07, P = 0.002), male sex (AOR = 5.02, P < 0.001), MAP (AOR = 1.03, P = 0.03), HDL-C (AOR = 1.04, P = 0.06) and hs-CRP (AOR = 1.06, P = 0.045) as well as TOS (AOR = 1.16, P = 0.03) and TAC (AOR = 1.51, P = 0.07) increased and GPX activity (AOR = 0.51, P = 0.07) decreased the odds of having CAD. Male sex (adjusted β (Aβ) =22.04, P < 0.001), age (Aβ = 0.87, P = 0.003), hs-CRP (Aβ = 0.72, P = 0.045) and TOS (Aβ = 2.75, P = 0.003) were associated with higher Gensini scores. Higher GPX activity (Aβ = −10.95, P = 0.03) was associated with lower Gensini scores.

**Conclusion:**

Biomarkers of oxidative stress, including TOS, TAC, and GPX activity, were associated with the presence, extent or severity of coronary atherosclerosis while no associations were observed for E-DII and DAQS scores.

## Introduction

The leading cause of death and loss of disability-adjusted life years (DALYs) worldwide is cardiovascular disease (CVD), particularly coronary artery disease (CAD) [1,2]. Globally, the prevalence of CVD has increased from approximately 271 million to 523 million in the last 30 years (1990–2019) [3]. In 2016, CAD accounted for 32.7% of the global burden of CVD and 2.2% of the total global burden of disease, as estimated by the Global Burden of Disease (GBD) research [4]. Much of this burden falls on low- and middle-income countries, accounting for nearly 7 million deaths and 129 million DALYs annually [1].

CAD has a chronic inflammatory nature [5]. Among the modifiable risk factors, unhealthy dietary habits such as high-calorie, nutrient-sparse diets have potent pro-inflammatory effects and are prominently involved in CVD [6]. Prolonged unhealthy diets result in gastrointestinal tract dysbiosis [7], dyslipidemia [8], increased production of pro-inflammatory cytokines, and endothelial dysfunction [6], all of which may play a role in CVD. Excessive food consumption, especially energy-dense foods, leads to increased energy intake exceeding energy expenditure. This metabolic imbalance causes the reduction of oxygen to a free radical, the superoxide ion [9]. There is a well-defined relationship between exposure to free radicals/elevated oxidative stress and CAD [10].

The dietary inflammatory index (DII^®^) is one of the primary approaches to describe anti/pro-inflammatory potential of diet. The DII was developed to quantify the inflammatory potential of an individual’s diet based on 45 food parameters identified in the literature as having sufficiently robust associations with six inflammatory biomarkers: interleukins (IL)-1β, −4, −6, −10, tumor necrosis factor-alpha (TNFα), and C-reactive protein (CRP) [11]. Many studies have demonstrated a direct relationship between higher DII and cardiovascular and other non-communicable disease rates and mortality risk [12–14]. Additionally, a higher DII, was associated with lower brachial artery flow-mediated dilation and higher cardiometabolic risk score [15]. A large cross-sectional study also found an association between DII and the prevalence of peripheral arterial disease [16]. Although many studies have shown an association between DII and cardiovascular outcomes, some others have failed to confirm it [17–19]. The dietary antioxidant quality score (DAQS) is another dietary score developed to estimate dietary intake of antioxidant nutrients [20]. The higher DAQS has a protective effect on the risk of cardiovascular disease and atherosclerosis [21].

There is a paucity of studies on the association between dietary anti-inflammatory and antioxidant potential and the risk and severity of CAD in non-Western populations, especially by documenting the disease using a gold standard method such as coronary angiography. Thus, the aim of this study was to investigate the association between dietary anti-inflammatory and antioxidant potential and some parameters of systemic inflammatory and oxidative status with the risk and severity of CAD based on coronary angiography.

## Methods

### Study population

A total of 158 candidates for coronary angiography were recruited into this cross-sectional study conducted at a tertiary center of excellence for cardiovascular disease. Inclusion criteria for the study were: age over 18 years and being a candidate for diagnostic coronary angiography. Exclusion criteria were: body mass index (BMI) <18.5 kg/m^2^; no significant changes to their dietary habits or following any specific diet or intentional weight change in the past three months; regular use of dietary supplements in excess of the recommended daily allowance in the past three months; or a history of CAD, heart failure, atrial fibrillation, acute coronary syndrome, severe valvular heart disease, cardiomyopathy, myocardial infarction, stroke, and coronary artery interventions, diabetes, thyroid dysfunction, major infection, trauma or major surgery in the past three months, organ failure, cancer, immune system defects, and skeletal muscle disease. In addition, as confirmed at recruitment, none of the participants in this study were smokers or substance abusers.

This study used data from an archived dataset and blood samples from a cross-sectional study of individuals undergoing diagnostic coronary angiography, in which various clinical, dietary, and biochemical parameters, as well as blood samples, were collected. All participants provided written informed consent at the time of enrollment, permitting the use of their anonymized data for research purposes. Data for the current study were accessed in May 2022 without identification of individual participants. The study protocol was approved by the institutional bioethics committee of Tehran University of Medical Sciences with approval ID “IR.TUMS.MEDICINE.REC.1400.370”.

### Data collection

#### Angiographic data.

Coronary angiography is considered the gold standard for the diagnosis of CAD [22]. Angiography was performed by cardiologists according to established protocols. The clinical criterion for CAD was defined as the presence of more than 50% stenosis in at least one of the main coronary arteries [23]. To determine the extent of disease, vessels were scored and classified according to the number of vessels involved (one, two, or ≥ three vessels). The severity of CAD was classified based on the number of vessels involved (single to multi-vessel) and the Gensini score [24]. The Gensini score is a widely used scoring method for assessing atherosclerosis on angiography. It provides information on the degree of stenosis and the location of the lesions in the coronary arteries. The Gensini score assigns a non-linear score to each lesion according to the severity of the stenosis, which is determined by the decrease in the diameter of the vessel lumen. A score of zero indicates the absence of coronary artery stenosis. In addition, a coefficient is used for each lesion based on the functional importance of each area in the coronary network. The final Gensini score is the sum of the scores for all the different lesions.

#### Anthropometric measurements.

Anthropometric measurements, including standing height, weight and waist circumference, were assessed before angiography, with participants wearing minimal clothing and barefoot. BMI [weight (kg)/height (m)^2^] and waist-to-hip ratio (WHR) were then calculated using standard formulas. Indirect assessment of body fat percentage based on the validated Deurenberg formula and visceral adiposity index (VAI) were calculated according to standard definitions described elsewhere [25].

#### Assessment of dietary intake.

Three 24-hour food diaries were collected by a trained dietitian on non-consecutive days, including two weekdays and one weekend day, to account for daily variations in dietary intake. These recalls were conducted through face-to-face interviews by a trained dietitian before the angiography procedure. Data from food recall forms were analyzed using the NUTRITIONIST IV (version 7.0; N-Squared Computing, Salem, OR, USA) software, which has been adapted for some local foods. This software calculates macro- and micro-nutrient content based on standardized food composition tables. The 3-day mean values for each 30 items were calculated and then the extracted nutrient data were then used to compute the dietary inflammatory index (DII) and dietary antioxidant quality score (DAQS) according to established methodologies.

#### Calculation of the DII and the DAQS.

The DII was calculated using data obtained from reported dietary intakes. Dietary intakes were obtained from the 3-day food recalls and extracted using NUTRITIONIST IV (version 7.0; N-Squared Computing, Salem, OR, USA). The calculation was based on a global comparative database derived from 11 populations across different countries, with inflammatory and antioxidant properties of food components determined from the literature [11]. To create a Z-score, the standard mean for each parameter was subtracted from the actual intake and divided by its standard deviation. These Z-scores were then converted into proportions (ranging from 0 to 1) and centred on 1 by doubling the value and subtracting 1. This transformation helped normalize the data and prevent skewness. The centred percentile value for each food parameter was then multiplied by its respective overall food parameter score to obtain the food parameter-specific DII score. Finally, all individual food parameter-specific DII/E-DII scores were summed to derive the final DII and E-DII scores. In the current study, data were available for 30 of the possible 45 food parameters including total energy, protein, carbohydrate, total fat, saturated fat, MUFA, PUFA, omega-3 fatty acids, omega-6 fatty acids, cholesterol, total fibre, iron, zinc, magnesium, selenium, vitamin A, beta-carotene, vitamin E, vitamin C, vitamin D, vitamin B1, vitamin B2, vitamin B3, vitamin B6, vitamin B9, vitamin B12, garlic, onion, green/black tea, caffeine. To remove the strong influence of energy intake on the results, energy-adjusted DII (E-DII^TM^) scores, were calculated with 29 food parameters (energy was in the denominator) using the density approach by calculating DII per 1000 kcal consumption. It uses the same scoring methodology. However, it is based on an energy-adjusted global comparative database [26]. The DII and E-DII provide a scale from maximally anti-inflammatory (most negative score) to maximally pro-inflammatory (most positive score). These scores have a potential range from approximately −9 to +8; i.e., from minimally to maximally pro-inflammatory, respectively.

To measure the antioxidant quality of the diet, intakes of five key micronutrients, including zinc, selenium, vitamin A, vitamin E, and vitamin C, were assessed and compared with the recommended daily allowance. A score of 0 was given for intakes below 2/3 times the RDA, and a score of 1 was given for intakes above this threshold. The final score ranged from 0 (indicating very poor diet quality) to 5 (indicating high diet quality) [27,28].

#### Biochemical measurements and other variables.

Before angiography, a venous blood sample was taken after a fasting period of 10–12 hours. After clotting, the collected samples were centrifuged at 3000 rpm for 20 minutes. The isolated serum was stored at −80°C until the further evaluation. Serum levels of fasting blood glucose (FBG), insulin, lipid profiles including low-density lipoprotein cholesterol (LDL-C), high-density lipoprotein cholesterol (HDL-C), total cholesterol and triglycerides, high-sensitivity (hs)-CRP, were measured using standard biochemical methods. All biochemical measurements were performed according to the instructions of the respective kits. Non-HDL-C cholesterol was calculated as total cholesterol – HDL-C. Triglyceride-FBG (TyG) index was calculated as (Ln [TG (mg/dL) × FBG (mg/dL)]/2).

Serum levels of malondialdehyde (MDA), total oxidant status (TOS), glutathione peroxidase (GPX) activity, and total antioxidant capacity (TAC) were assessed using commercial kits (Novin Navand Salamat Pishtaz Co., Urmia, Iran). Oxidative stress index (OSI) was calculated as TOS/ TAS × 100. The homeostatic model assessment for insulin resistance (HOMA-IR) was calculated according to the following formula: HOMA-IR = fasting glucose (mg/dl) × fasting insulin (μU/mL)/ 405. Mean arterial pressure (MAP) was calculated as 1/3 systolic + 2/3 diastolic blood pressure.

### Statistical analyses

A binary logistic regression model was used to assess the association of E-DII score, markers of oxidative stress and other cardiovascular risk factors with CAD and in 3 vessel disease (3VD) compared with 1VD. Multiple binary logistic regression was used to account for the role of potential confounders including age, sex, and BMI on these associations. General linear models were used to assess the relationships of quantitative or binary variables with the Gensini score and to adjust for the effect of age, sex, and BMI on these associations. Multiple binary logistic regression and general linear models with LR backward model selection method were used to identify the main determinants of having CAD, having 3VD, and Gensini score, presented with adjusted odds ratio (AOR) or adjusted regression coefficient (Aβ), 95% confidence interval (CI) and P values. All statistical analyses were performed using SPSS (IBM SPSS Statistics for Windows, IBM Corp., Version 22.0. Armonk, NY, USA). P values less than 0.05 were considered statistically significant.

## Results

There was no statistically significant relationship between E-DII or DAQS with CAD in the baseline crude and adjusted models. After adjustment for confounding variables, an increase in FBG (AOR = 1.02, 95% CI:1.003–1.04, P = 0.02), HDL-C (AOR = 1.04, 95% CI: 1.002–1.079, P = 0.04), MAP (AOR = 1.028, 95% CI:1.002–1.055, P = 0.04) and age (AOR = 1.06, 95% CI:1.021–1.100, P = 0.002) level increased the odds of developing CAD, whereas being female (AOR = 0.295, 95% CI:0.132–0.660, P = 0.003) decreased the odds of having CAD. No correlations were found between other variables and the odds of having CAD in basic models (Table 1). There were no significant differences in using aspirin (64%.vs 68.1%, p = 0.588) or statins (47.7% vs. 61.1%, p = 0.092) in patients without CAD compared to those with CAD.

**Table 1:** Association of dietary indices, markers of inflammation and oxidative stress and other cardiometabolic risk factors with CAD.

Variables	CAD (-) (N = 86)		CAD (+) (N = 72)		Simple logistic regression					
					OR	95% CI		P ^¥^	Adjusted P	
	Mean	SD	Mean	SD		Lower	Upper		P^¥^**	P^¥^***
E-DII	1.7	1.1	1.6	1.1	0.92	0.70	1.22	0.61	0.62	0.57
DAQS	3.0	1.0	3.0	1.0	0.83	0.65	1.06	0.15	0.14	0.14
MDA (nmol/ ml)	61.8	24.9	57.0	22.6	0.99	0.97	1.00	0.13	0.14	0.21
TOS (µmol Equiv./L)	3.4	2.5	4.0	3.4	1.01	0.93	1.09	0.72	0.47	0.09
GPX (mU/mL)	9.6	0.5	9.5	0.6	0.73	0.41	1.31	0.30	0.43	0.47
TAC (mmol Fe^2 + /^L)	1.3	0.8	1.5	0.8	1.37	0.90	2.07	0.14	0.06	0.07
OSI	312.6	264.9	306.8	263.9	1.00	0.99	1.001	0.89	0.78	0.71
Hs-CRP (mg/l)	3.4	5.5	4.6	9.1	1.02	0.97	1.07	0.31	0.11	0.11
HOMA-IR	3.5	2.1	4.1	2.8	1.11	0.97	1.27	0.11	0.51	0.06
FBG (mg/dl)	99.5	14.8	106.2	20.0	1.02	1.00	1.04	0.02	0.02	0.02
TG (mg/dl)	140.4	64.7	146.7	76.2	1.00	0.99	1.00	0.57	0.18	0.19
LDL-C (mg/dl)	90.5	34.3	92.0	34.6	1.00	0.99	1.01	0.79	0.47	0.49
HDL-C (mg/dl)	40.3	9.5	42.5	9.9	1.02	0.99	1.05	0.16	0.047	0.04
Cholesterol (mg/dl)	155.5	39.4	158.4	44.2	1.00	0.99	1.00	0.67	0.26	0.27
Non-HDL-C (mg/dl)	115.0	35.4	115.9	41.6	1.00	0.99	1.01	0.89	0.45	0.47
TyG index	8.7	0.5	8.8	0.6	1.37	0.73	2.58	0.32	0.07	0.07
MAP (mmHg)	90.0	12.8	94.9	14.9	1.03	1.00	1.05	0.03	0.02	0.04
Age (year)	56.7	9.9	60.4	9.1	1.04	1.00	1.07	0.02	<0.001	<0.001
Sex (female) (N, %)	39.0	45.3	19.0	26.4	0.43	0.22	0.84	0.02	<0.001	<0.001
Weight (kg)	79.6	12.4	79.2	18.1	0.99	0.97	1.01	0.87	0.74	–
Waist (cm)	102.7	10.7	101.9	15.3	0.99	0.97	1.02	0.70	0.77	0.73
BMI (kg m^ − 2^)	30.0	4.9	29.4	6.7	0.98	0.92	1.03	0.49	0.59	–
BF (%)	37.6	10.4	35.8	11.0	0.98	0.95	1.01	0.27	0.60	–
VAI	2.7	1.7	2.5	1.5	0.91	0.74	1.13	0.43	0.78	0.83
WHR (Per 0.1 increased ratio)	9.5	0.5	9.7	0.7	1.69	0.98	2.90	0.06	0.12	0.13

*Data have been presented as mean, SD or N, percent.

¥ Based on Binary logistic.

**p Adjusted for age and sex.

*** p Adjusted for age, sex, and BMI.

In the final step, the logistic regression model showed that age, sex, MAP, hs-CRP, HDL-C, TOS, GPX, and TAC were the most important determinants of incident CAD. Male participants were significantly more likely to have CAD than female participants (AOR = 5.02, 95% CI: 2.09–12.04, P < 0.001). Age (AOR = 1.07, 95% CI: 1.03–1.12, P = 0.002), hs-CRP (AOR = 1.06, 95% CI: 1–1.12, P = 0.045), MAP (AOR = 1.03, 95% CI: 1–1.06, P = 0.03), HDL-C (AOR = 1.04, 95% CI: 0.10–1.08, P = 0.06), TOS (AOR = 1.16, 95% CI: 1.02–1.32, P = 0.028), and TAC (AOR = 1.51, 95% CI: 0.97–2.35, P = 0.07) increased the odds of having CAD, whereas GPX (AOR = 0.51, 95% CI: 0.25–1.05, P = 0.07) decreased the odds of having CAD.

Binary regression analyses showed no statistically significant associations between E-DII or DAQS and extent of CAD, even after adjustment for age, sex, and BMI. Higher levels of TOS (AOR = 3.033, 95% CI:1.33–6.90, P = 0.008), HOMA-IR (AOR = 1.75, 95% CI:1.119–2.737, P = 0.01), triglycerides (AOR = 1.019, 95% CI:1.003–1.035, P = 0.02), TyG index (AOR = 20.54, 95% CI:2.091–201.95, P = 0.01), VAI (AOR = 5.89, 95% CI:1.63–21.31, P = 0.007) and OSI (AOR = 1.014, 95% CI:1.004–1.025, P = 0.008) were associated with higher odds of having 3VD compared with 1VD, whereas female sex (AOR = 0.13, 95% CI:0.021–0.821, P = 0.03) was associated with lower odds of having 3VD after adjusting for age, sex, and BMI (Table 2). The final logistic regression model showed that sex, BMI, HDL-C, TOS, and TyG index were the most important determinants of having 3VD compared to 1VD. Female participants had significantly lower odds of having 3VD compared to male participants (AOR = 0.14, 95% CI: 0.01–1.64, P = 118). HDL-C (AOR = 0.85, 95% CI: 0.74–0.98, P = 0.027) and BMI (AOR = 0.76, 95% CI: 0.61–0.96, P = 0.018) decreased the odds of having 3VD, whereas TOS (AOR = 1.6, 95% CI: 0.8–3.2, P = 0.185) and TyG index (AOR = 13.7, 95% CI: 0.56–342.46, P = 0.11) increased the odds of having 3VD.

**Table 2:** Association of dietary indices, markers of inflammation and oxidative stress and other cardiometabolic risk factors with extent of CAD.

Variables	CAD (+) (N = 72)						Simple logistic regression					
	1VD (N = 25)		2VD (N = 28)		3VD (N = 19)		OR	95% CI		P ^¥^	Adjusted P	
	Mean	SD	Mean	SD	Mean	SD		Lower	Upper		P^¥^**	P^¥^***
E-DII	1.5	1.0	1.6	1.2	1.6	1.1	1.09	0.61	1.94	0.78	0.69	0.65
DAQS	3.0	1.0	3.0	1.0	3.0	1.0	1.00	0.65	1.55	1.00	0.82	0.93
MDA (nmol/ ml)	56.8	21.7	53.8	22.1	61.9	24.6	1.01	0.98	1.04	0.46	0.60	0.66
TOS (µmol Equiv./L)	2.5	1.3	4.8	4.0	5.0	4.0	1.59	1.04	2.44	0.03	0.01	0.01
GPX (mU/mL)	9.4	0.8	9.6	0.4	9.5	0.6	1.34	0.53	3.41	0.54	0.39	0.50
TAC (mmol Fe^2 + /^L)	1.6	1.0	1.5	0.9	1.4	0.5	0.73	0.33	1.65	0.45	0.51	0.68
OSI	210.0	140.4	356.8	340.4	360.3	234.1	1.005	1.001	1.008	0.024	0.006	0.008
Hs-CRP (mg/l)	3.8	7.2	4.1	7.9	6.3	12.7	1.03	0.96	1.09	0.42	0.37	0.38
HOMA-IR	3.8	2.9	4.4	2.5	4.2	3.0	1.05	0.85	1.29	0.67	0.37	0.01
FBG (mg/dl)	101.6	16.9	111.0	23.4	105.3	17.5	1.01	0.98	1.05	0.47	0.65	0.40
TG (mg/dl)	128.6	62.3	158.0	88.6	154.4	73.2	1.01	1.00	1.02	0.21	0.07	0.02
LDL-C (mg/dl)	94.8	33.8	86.1	29.0	97.0	43.1	1.00	0.99	1.02	0.85	0.54	0.36
HDL-C (mg/dl)	45.5	10.4	41.0	10.2	40.8	8.1	0.95	0.89	1.01	0.12	0.22	0.15
Cholesterol (mg/dl)	159.4	40.7	151.8	36.7	166.7	57.7	1.00	0.99	1.02	0.61	0.24	0.14
Non-HDL-C (mg/dl)	113.9	39.2	110.8	36.2	125.9	51.6	1.01	0.99	1.02	0.38	0.13	0.07
TyG index	8.7	0.5	8.9	0.6	8.9	0.5	2.40	0.69	8.32	0.17	0.05	0.01
MAP (mmHg)	97.2	12.8	94.2	15.4	92.8	17.0	0.98	0.94	1.02	0.33	0.47	0.85
Age (year)	59.5	8.8	60.3	10.0	61.6	8.4	1.03	0.96	1.11	0.42	0.18	0.29
Sex (female) (N,%)	12	48	5	17	2	10.5	0.13	0.02	0.67	0.02	0.01	0.03
Weight (kg)	83.5	19.6	78.6	16.4	74.4	18.3	0.97	0.94	1.01	0.13	0.23	–
Waist (cm)	106.5	16.2	100.7	14.1	97.4	14.9	0.96	0.92	1.00	0.07	0.22	0.67
BMI (kg m^ − 2^)	31.1	6.8	29.5	6.8	26.9	5.7	0.89	0.80	1.00	0.046	0.24	–
BF (%)	40.0	10.8	35.0	11.6	31.3	8.7	0.91	0.85	0.98	0.01	0.24	–
VAI	2.2	1.3	2.7	1.8	2.4	1.0	1.11	0.66	1.88	0.68	0.04	0.01
WHR (Per 0.1 increased ratio)	9.7	0.8	9.7	0.6	9.7	0.7	0.96	0.43	2.14	0.92	0.86	0.60

*Data have been presented as mean, SD or N, percent.

¥ Based on Binary logistic.

**p Adjusted for age and sex.

*** p Adjusted for age, sex, and BMI.

There was no statistically significant relationship between either E-DII or DAQS score and Gensini score. After adjusting for the likely effects of age, sex, and BMI, changes in TOS (Aβ = 2.055, 95% CI: 0.369–3.741, P = 0.02), age (Aβ = 0.804, 95% CI: 0.266–1.342, P < 0.001), sex (Aβ = −19.78, 95% CI: −31.55–8.008, P < 0.001), and WHR (Aβ = 9.62, 95% CI: 0.711–18.53, P = 0.03) were found to be associated with changes in Gensini score. The use of aspirin (p = 0.837) and statins (p = 0.299) had no significant correlation with the Gensini score. No significant associations were observed between other variables and the Gensini score in the baseline models (Table 3). However, the final general linear model from the backward selection model showed that age, sex, hs-CRP, TOS, and GPX were the main determinants of the Gensini score. Being male was associated with an increase in Gensini score of approximately 22-unit (Aβ = 22.04, 95% CI: 10.92–33.15, P < 0.001). Each unit increase in age (Aβ = 0.87, 95% CI: 0.31–1.43, P = 0.003), hs-CRP (Aβ = 0.72, 95% CI: 0.02–1.43, P = 0.045) and TOS (Aβ = 2.75, 95% CI: 0.93–4.57, P = 0.003) was associated with approximately about 0.8, 0.7, and 2.8 units increase in Gensini score, respectively. Each unit increase in GPX activity was associated with approximately 11 units decrease in Gensini score (Aβ = −10.95, 95% CI: −20.71- −1.19, P = 0.028).

**Table 3:** Association of dietary indices, markers of inflammation and oxidative stress and other cardiometabolic risk factors with the Gensini score.

Variable	Simple general linear model				Adjusted P	
	Beta	95% CI		P^ƚ^		
		Lower	Upper		P^ƚ^ **	P^ƚ^ ***
E-DII	−2.30	−7.04	2.45	0.34	0.29	0.26
DAQS	−1.78	−5.76	2.26	0.39	0.38	0.41
MDA (nmol/ ml)	−0.07	−0.29	0.16	0.56	0.53	0.56
TOS (µmol Equiv./L)	1.75	−0.03	3.52	0.05	0.02	0.02
GPX (mU/mL)	−6.73	−16.17	2.72	0.16	0.25	0.25
TAC (mmol Fe^2 + /^L)	3.39	−3.25	10.03	0.32	0.20	0.18
OSI	0.001	−0.02	0.02	0.90	0.53	0.53
Hs-CRP (mg/l)	0.36	−0.36	1.08	0.33	0.11	0.10
HOMA-IR	1.20	−0.98	3.39	0.28	0.17	0.08
FBG (mg/dl)	0.20	−0.10	0.50	0.19	0.20	0.16
TG (mg/dl)	0.01	−0.06	0.09	0.72	0.25	0.21
LDL-C (mg/dl)	0.04	−0.12	0.19	0.66	0.38	0.36
HDL-C (mg/dl)	−0.03	−0.58	0.51	0.91	0.59	0.62
Cholesterol (mg/dl)	0.03	−0.10	0.15	0.69	0.25	0.22
Non-HDL-C (mg/dl)	0.03	−0.11	0.18	0.63	0.25	0.22
TyG index	3.92	−6.67	14.50	0.47	0.10	0.06
MAP (mmHg)	0.25	−0.13	0.63	0.20	0.15	0.14
Age (year)	0.56	0.02	1.10	0.04	< 0.001	< 0.001
Sex	−16.89	−27.60	−6.18	< 0.001	< 0.001	< 0.001
Weight (kg)	−0.15	−0.49	0.20	0.42	0.67	–
Waist (cm)	−0.21	−0.61	0.20	0.33	0.84	0.88
BMI (kg m^ − 2^)	−0.77	−1.69	0.15	0.10	0.76	–
BF (%)	−0.54	−1.03	−0.04	0.34	0.72	–
VAI	−1.40	−4.82	2.03	0.43	0.66	0.61
WHR (Per 0.1 increased ratio)	9.26	0.78	4.58	0.03	0.08	0.03

ƚ Based on GLN.

** Adjusted for age for sex.

*** Adjusted for age, sex, and BMI.

## Discussion

In this study, no association was observed between the E-DII and the DAQS scores and the presence or severity of CAD. Factors such as age, male sex, MAP, HDL-C and hs-CRP along with oxidative stress markers such as TOS, TAC were directly associated and GPX activity was inversely associated with the presence of CAD. When comparing 3VD to 1VD, female sex, BMI, HDL-C were inversely and TOS and TyG index were directly important determinants of CAD extent. Male sex, age, hs-CRP and TOS have contributed to higher, whereas higher GPX activity was associated with lower Gensini scores.

The association between the DII/E-DII and CAD has been investigated in many studies. Higher DII scores and a pro-inflammatory diet may increase the risk of developing CVD through mechanisms involving inflammation [11,29], expression of adhesion molecules including selectins and cadherins [30], insulin resistance [31], and unfavorable lipoprotein profiles [12,32,33]. The SUN cohort in Spain, which is one of the largest studies in this field, showed an approximately two-fold higher CVD risk when comparing the highest vs. lowest quartiles of DII score [34]. A comprehensive study of NHANES III data found that participants with a more pro-inflammatory diet, as indicated by higher DII scores, had an increased risk of CVD mortality [35]. Another article based on NHANES 2007–2011 data showed a significant direct association between DII score and CVD risk estimated from the Framingham risk score [36].

However, similar to the current results, not all available studies have reported a positive association between the DII and CAD. The MASHAD cohort study, also conducted in Iran, and which included 4,672 participants with and without CVD, found no significant association between DII score and the risk of total CVD, myocardial infarction, stable and unstable angina [17]. In a cross-sectional study of coronary artery bypass grafting (CABG) candidates, no significant differences in the number of diseased vessels, New York Heart Association functional class, and left ventricular ejection fraction were observed across DII categories [37]. Additionally, a meta-analysis study supported the association of DII with increased risk of CVD and CVD mortality. However, in a subgroup analysis that included a small number of studies, there was no association between higher DII and ischemic heart disease other than myocardial infarction [13].

Limited data are available on the association between DII/E-DII and the severity of CAD. A recent study showed a significant increase in the risk of severe CAD with the highest versus lowest tertiles of the DII (OR 3.7 and AOR 6.1) [38]. Metabolic diseases such as diabetes may contribute to the association between CAD and dietary factors, which were not excluded in the aforementioned study. Interestingly, the prevalence of diabetes and fatty liver decreased significantly from the lowest to the highest tertile of the DII score. These changes were in the opposite direction with increasing Gensini score and CAD severity and were not included in the adjusted models. Additionally, a Gensini score > 20 was considered severe CAD, which is not a widely accepted cut-off and was not also seen in our data set.

The inconsistencies in the association of DII and CAD between studies might be due to several reasons. Probably one of the most important contributors is the method of disease documentation; as the majority of studies have considered a history of myocardial infarction, angina, stroke, or coronary heart disease as the definition of CVD or CAD rather than using the gold standard methods such as coronary angiography. This is also the reason why very few studies have reported the association between DII and CAD severity based on Gensini or other severity scores. Inclusion of participants with a recognized history of CAD versus those undergoing a diagnostic technique to detect CAD might also affect the true association of dietary scores with the disease. This is because patients with CAD or other diseases may change their dietary habits on the recommendation of their health care providers. This dietary change may potentially mask the pro-inflammatory effects of the baseline diet, resulting in a null association between the DII and CAD. Alternatively, the lack of association may indicate reverse causality, in which individuals with existing CAD may have already modified their diet in response to their health condition. In other words, the time frame of exposure, disease development, disease recognition and data collection may contribute to the inconsistencies in findings across studies. Risk factors for chronic diseases including CAD, typically act over a long period of time before culminating in the development of disease. Therefore, the lack of association between the DII and CAD does not rule out the effect of a pro-inflammatory diet on CAD and highlights the importance of considering the duration and timing of exposure when collecting and interpreting data.

Methodological differences, such as study design, data collection, analytical approaches and sample size, which may affect statistical power, also contribute to the inconsistencies in study results. Differences in demographic characteristics, presence of other diseases and health problems and comorbidities (including non-clinically evident disease), genetic predisposition, and lifestyle factors may also contribute to the observed variations in the association between the DII and CAD. It is important to note that the dietary data collected over three days may not be sufficient to detect associations with disease states that may take several years, or even decades, to manifest. Second, although the DII consists of 45 food parameters, 30 parameters were used in our study due to limitations such as unavailability in food recalls or food composition tables. Nevertheless, previous studies that did not include parameters similar to those missing in our study have shown that reducing the number of parameters does not affect the discriminatory potential of the DII. In addition, it would probably be valuable to reconsider the use of commonly developed scores such as DII and DAQS in favor of disease-specific versions that take into account some of the most important disease-related determinants.

The results of this study showed that TOS was directly associated with having CAD, having 3VD, and having a higher Gensini score. TAC was also associated with the presence of CAD, but not with the extent or severity of the disease. In contrast, GPX activity, one of the major antioxidant enzymes, was inversely associated with CAD and Gensini score. The association of oxidative stress with CVD is well established. However, the relationship between TOS and CAD may be influenced by TAC, the specific oxidants measured, and the method used to calculate TOS. The optimal TOS and TAC levels for CAD prevention may vary depending on individual characteristics and other covariates. We did not observe an association between OSI and CAD despite higher TAC levels in the patient group. Aydin et al. also found higher TAC levels in patients with higher Gensini scores. However, the OSI did not differ significantly between the study groups [39]. The use of OSI has also been proposed in other studies [40]. Changes in antioxidant capacity and higher TAC might be a compensatory mechanism in patients with CAD. It could also be due to other factors such as the use of treatments with anti-oxidant properties such as statins and ACE inhibitors.

GPX activity has been shown to have a potential role in reducing the extent and severity of CAD. Alternatively, it could be the effect of the disease on the oxidant/anti-oxidant balance. However, this relationship is complex and influenced by factors such as glutathione levels and specific GPX isoforms. In some studies, lower glutathione levels in advanced CAD were associated with oxidative stress, with GSH-Px activity inversely correlated with the number of stenotic coronary artery branches, suggesting a protective role of GSH-Px in CAD [41–43]. Another study suggested that higher glutathione levels are observed in those with more advanced CAD, possibly as an adaptive response to increased oxidative stress [44]. These studies present conflicting results suggesting that GSH levels may not consistently correspond to GPX activity in CAD patients. Additionally, the specific GPX isoforms, such as GPX1, GPX3, and GPX4, and the method used to measure GPX activity should be considered, as they may have different functions and distributions in tissues and potentially different effects on CAD characteristics.

TyG index, a recently studied reliable, non-invasive, and effective surrogate of insulin resistance, was associated with higher odds of 3VD compared to 1VD with a substantial OR of 13.7. Other recent studies have also shown a direct correlation between the TyG index and the Gensini score [45], coronary calcification and plaque burden > 70% [46], SYNTAX score [47], higher incidence of CAD and carotid atherosclerotic plaque [48]. This simple and inexpensive index could be part of the risk assessment in patients with CVD. The current study also showed an association between CAD and/or CAD severity and some of the traditional risk factors. Hs-CRP was directly associated with higher CAD risk and higher Gensini scores. While some studies suggested hs-CRP as a useful indicator of atherosclerotic plaque instability and inflammatory response activity in CAD [49], others found a negligible and statistically non-significant correlation between hs-CRP and CAD severity [50]. The effect of hs-CRP on CAD severity should be interpreted considering indicators of lipid metabolism, types of CAD outcomes and methods of measurement. In addition, optimal hs-CRP levels for CAD prevention may vary according to individual characteristics and genetic predisposition.

Male gender was associated with a higher risk of CAD and CAD extent and severity. The association between sex and CAD is multifactorial. Sex chromosome and hormonal mechanisms interact, and the exposure to endogenous estrogens during the fecundable period in women delays the manifestation of atherosclerotic disease [51]. Estrogens have regulatory effects on lipids, inflammatory markers, coagulation factors, and promote direct vasodilation. These hormonal differences contribute to sex differences in CAD risk [52]. Older age was associated with having CAD and higher Gensini scores. Increased oxidative stress, inflammation, and endothelial dysfunction with aging affect vascular function and structure. In addition, the interaction between age and other risk factors such as diabetes, hypertension, and dyslipidemia may influence the progression of CVD. In the current study, higher MAP was associated with an increased risk of CAD but not with the extent and severity of CAD. This association may be confounded by other health conditions. In a study of CAD patients with hypertension, both low and high diastolic blood pressure were associated with an increased risk of CAD. Pulse pressure (PP) has also been identified as a strong predictor of cardiovascular events [53].

HDL-C levels were associated with a higher risk of CAD in this study. This association was weak in the final regression model (OR 1.04). Some studies have shown that both low and high levels of HDL-C are associated with an increased risk of CVD. HDL-C levels above 80 mg/dL were associated with a higher risk of all-cause and cardiovascular death, especially in populations with CVD [54]. However, in our study the mean values of HDL-C were 40.3 vs. 42.5 mg/dl in non-CAD participants compared to CAD patients, which is not clinically relevant and is within the normal ranges. Differences in the composition and properties of HDL-C particles may also influence the development of CAD. When the analysis was restricted to the extent of CAD, the mean values of HDL-C had a decreasing trend with 3VD, and HDL-C levels were inversely associated with disease severity (OR 0.85). Other studies have found lower HDL-C levels in subjects with higher Gensini scores, suggesting a possible association between HDL-C and CAD severity [55].

This study had several limitations and strengths. First, due to the cross-sectional study design employed, causality cannot be established. Issues regarding reverse causality and self-selection into such a population are of concern and cannot be ruled out in this kind of study design. Thus, it is imperative to conduct longitudinal studies with large sample sizes to validate and confirm these findings. Also, it should be noted that the DII scores were generally higher (more proinflammatory) than what we see in most populations, including in Iran [56]. Additionally, the modest sample size of our study may have compromised the precision of the OR estimates, resulting in wider confidence intervals than those observed in studies with larger sample sizes. Nevertheless, careful recruitment strategies were used to ensure that the sample was representativeness of the population studied and to minimize the confounding effects of important covariates such as diabetes. In addition, this study used multiple forms of analysis and implemented rigorous control measures to account for various confounding factors. Logistic regression and general linear models with backward selection were used to identify the primary determinants and key factors associated with the presence of CAD and the extent and severity of the disease. It is imperative that these limitations as well as developing disease-specific versions of dietary scores such as DII, be addressed in future research efforts to provide a more comprehensive understanding of the complex relationships among diet, inflammation, and disease status and outcomes.

## Conclusion

There were no significant associations between dietary inflammatory/antioxidant scores, DII and DAQS with the presence, severity and extent of CAD based on coronary angiography. Age, male sex, MAP, HDL-C, hs-CRP, TOS, and TAC were directly and GPX activity was inversely associated with the odds of having CAD. When comparing 3VD to 1VD, female sex, BMI, HDL-C were inversely and TOS and TyG index were directly associated with CAD extent. Male sex, age, hs-CRP and TOS have contributed to higher, whereas higher GPX activity was associated with lower Gensini scores. Further research is needed to better understand how useful novel nutritional indices are in predicting CAD.
